# Toward an ecologically meaningful view of resource stoichiometry in DOM-dominated aquatic systems

**DOI:** 10.1093/plankt/fbv018

**Published:** 2015-03-19

**Authors:** Martin Berggren, Ryan A. Sponseller, Ana R. Alves Soares, Ann-Kristin Bergström

**Affiliations:** 1 Department of Physical Geography and Ecosystem Science, Lund University, SE-223 62 Lund, Sweden; 2 Department of Ecology and Environmental Science, Umeå University, SE-901 87 Umeå, Sweden

**Keywords:** nutrient limitation, dissolved organic matter, bioavailability, bacterioplankton production, phytoplankton primary production, basal resource stoichiometry

## Abstract

Research on nutrient controls of planktonic productivity tends to focus on a few standard fractions of inorganic or total nitrogen (N) and phosphorus (P). However, there is a wide range in the degree to which land-derived dissolved organic nutrients can be assimilated by biota. Thus, in systems where such fractions form a majority of the macronutrient resource pool, including many boreal inland waters and estuaries, our understanding of bacterio- and phytoplankton production dynamics remains limited. To adequately predict aquatic productivity in a changing environment, improved standard methods are needed for determining the sizes of active (bioavailable) pools of N, P and organic carbon (C). A synthesis of current knowledge suggests that variation in the C:N:P stoichiometry of bioavailable resources is associated with diverse processes that differentially influence the individual elements across space and time. Due to a generally increasing organic nutrient bioavailability from C to N to P, we hypothesize that the C:N and N:P of bulk resources often vastly overestimates the corresponding ratios of bioavailable resources. It is further proposed that basal planktonic production is regulated by variation in the source, magnitude and timing of terrestrial runoff, through processes that have so far been poorly described.

## INTRODUCTION

The two most important production processes at the base of planktonic food chains, phytoplankton primary production (PP) and bacterioplankton secondary production (BP), are strongly influenced by loading of nutrients from land (Jansson *et al.*, 2000; Hitchcock and Mitrovic, 2013). In many aquatic systems with a high natural input of land-derived nutrients, for example brown-water lakes, PP and BP are often equally important production processes at the base of planktonic food chains, supporting fluxes of energy and matter to higher trophic levels (Karlsson *et al.*, 2002). These nutrients may be supplied in either inorganic mineral form or they may be chemically bound to dissolved organic matter (DOM, i.e. “organic” nutrients).

There is a long tradition of studies that explain how anthropogenic increases in inorganic nutrient concentrations enhance PP (e.g. Schindler, 1977) and BP (Pace and Cole, 1996), but the impact on these processes of organic nutrient inputs from land is more uncertain. Nonetheless, fresh and coastal waters dominated by organic forms of carbon, nitrogen and phosphorus (DOC, DON and DOP, respectively) are globally widespread, particularly in regions not subject to elevated anthropogenic inputs of inorganic nutrients (Perakis and Hedin, 2002; Stepanauskas *et al.*, 2002). Moreover, systems in which DOM from land forms a majority of the total nutrient pools, hereafter called “DOM-dominated” systems, are likely to become increasingly abundant with the current trends of rising DOM concentrations in temperate and boreal continental water systems (Monteith *et al.*, 2007).

An obstacle to studies of these DOM-dominated systems is that no standard nutrient analyses can determine the sizes of total bioavailable nutrient pools, that is, the nutrients that can be readily assimilated by phytoplankton and bacterioplankton. For example Jansson *et al.* (Jansson *et al*., 2012) found that none of the standard analyses of P (total phosphorus, TP; dissolved reactive phosphorus, DRP) came close to characterizing seasonal patterns of observed P bioavailability for bacterioplankton. Similarly, major components of the bioavailable DON and DOC are chemically undefined and, thus, cannot be predicted from known chemical analyses (Stepanauskas *et al.*, 2002; Berggren *et al.*, 2010a). Such deficiencies preclude an adequate understanding of natural nutrient control of planktonic communities in DOM-dominated systems, and therefore make the effects of anthropogenic nutrient loading as well as climate-driven changes in terrestrial runoff difficult to predict (Seitzinger and Sanders, 1997).

Here we introduce and discuss existing concepts related to plankton nutrition, resource stoichiometry and nutrient control over basal plankton production processes (PP and BP) in inland and estuarine waters. We then exemplify the pitfalls of applying these concepts to strongly land-influenced and DOM-rich waters, where organic nutrients from land provide the majority of bulk macronutrients to aquatic ecosystems. Finally, we discuss how the research field may be advanced through increased use of ecologically meaningful multi-element assessments of the bioavailability.

## NUTRIENTS AND PRODUCTIVITY: BACKGROUND TO THE FIELD

The basic building blocks of life, such as peptides, phospholipids and nucleic acids, are composed only of a few elements: the macronutrients. These are assimilated during phytoplankton growth in proportions roughly depicted by the empirical formula C_106_H_175_O_42_N_16_P (Redfield, 1958; Anderson, 1995). For comparison, heterotrophic bacterioplankton incorporates macronutrients at variable but often relatively low C:N:P proportions, ca. 50:10:1 (Fagerbakke *et al.*, 1996; Vrede *et al.*, 2002). Generally, up to 20 additional trace elements (micronutrients) are needed in most organisms to maintain cell functions, e.g. iron (Fe) and copper (Cu) used in electron transport chain proteins, manganese (Mn), boron (B) and zinc (Zn) that regulate various enzymes and so on (Raven *et al.*, 2013). However, the relative demand for these trace elements likely varies across major planktonic groups, e.g. high Fe and molybdenum (Mo) demand in diazotrophs (N fixers). Further, the need for certain elements may be highly species dependent, e.g. silicon (Si) needed to support the cell wall structure of diatoms.

### Who are the players?

In the face of eutrophication, the search for key nutrients that regulate aquatic productivity has drawn considerable scientific and societal attention, with much of the historical focus being on P and N. In Froelich's (Froelich, 1988) analogy between an aquatic ecosystem and a chess board, P is the king of all players, restricted in his movements yet dictating the final ecosystem outcomes. Indeed, there are empirical and theoretical grounds for the perspective that P controls aquatic productivity over the long time scales of ecosystem development (Sterner, 2008; Schindler, 2012). However, at any given moment, it is the queen, N, that controls much of the dynamics of the game (Froelich, 1988). Mounting experimental evidence now shows that N and P are often co-limiting in diverse aquatic environments (Harpole *et al.*, 2011) and that N exerts at least as much short-term control over PP and BP as does P in lakes (Faithfull *et al.*, 2011), estuaries and coasts (Howarth and Marino, 2006; Hitchcock and Mitrovic, 2013).

In terms of micronutrient impact, PP (and sometimes also BP) has been shown to respond positively to increases in Fe (Coale *et al.*, 1996; Arrieta *et al.*, 2004; Vrede and Tranvik, 2006), Mo, Co and Cu (Downs *et al.*, 2008). Nonetheless, it is generally P, N and organic C that constrain basal planktonic production in DOM-dominated waters (Jansson, 1998). Therefore, the focus here is on these three key macronutrients.

The role of C as a productivity-constraining nutrient is, however, less straightforward than those of N and P. In the classical microbial loop concept, plankton productivity is based on PP and, thus, on inorganic C (classically considered to be non-limiting), while BP only represents a share of the PP-derived detritus that cycles back into secondary biomass production (Azam *et al.*, 1983; Cole *et al.*, 1988). We now know that the C flux through bacterioplankton in both lakes (Jansson *et al.*, 2000) and estuaries (del Giorgio *et al.*, 1997) can be larger than what is possible to sustain by PP, and BP is often regulated by variations in the supply of land-derived DOM (Berggren *et al.*, 2009a). Further, in unproductive DOM-dominated systems mixotrophic algae and other bacterivores represent a phagotrophic C incorporation similar in magnitude to C produced via photosynthesis (Jansson *et al.*, 1999). Thus, the bioavailable organic C *per se* can act as a major resource that regulates bacterioplankton productivity (Berggren *et al.*, 2010b) and contributes to C transfer in the lake food chains (Karlsson *et al.*, 2012; Berggren *et al.*, 2014).

### The concept of nutrient bioavailability

To serve as a nutritional source for biota, molecules are required to have a chemical structure that allows them to be taken up and utilized by cells, i.e. they need to be “bioavailable.” Major parts of the potentially bioavailable N, P and organic C pools in inland waters are covalently bound or chelated to large and colloidal DOM molecules (Jones *et al.*, 1988) that do not pass bacterial or phytoplankton cell membranes (unless vesicle-transported through pinocytosis), yet such nutrients can be transformed into smaller assimilable molecules through the action of various extracellular or membrane-associated enzymes (Likens, 2010). An adequate definition of “bioavailable nutrients” must therefore include nutrients that can potentially be assimilated by a given plankton community or culture, either directly or facilitated by enzymatic processing (Stepanauskas *et al.*, 2002).

Given this inclusive definition, quantifying these pools requires the use of so-called bioassays, where biological nutrient uptake is measured in standardized environmental conditions. A general overview of the most common analytical approaches to bioavailability determination is presented in Table I, but it should be stressed that there is a plethora of operational bioavailability definitions in the literature, each associated with a specific methodology (Bronk *et al.*, 2007; Guillemette and del Giorgio, 2011). Unfortunately, very few studies to date have simultaneously measured the bioavailability of multiple macronutrients (Table II), most likely due to the lack of a methodological framework. For example, the widely applied isotope tracer techniques are nutrient-specific and not designed for comparisons of bioavailability among different elements (Table I).

**Table I:** Common methods used to measure bioavailability, especially of organic nutrients

Method	Time frame	Description	Strengths	Weaknesses	References
Δ concentration	From 1 week to ca. 3 months	Bioavailability is considered to be equal to the change in the bulk nutrient concentration, e.g. of DOC, DON, DOP or total dissolved P, measured during incubations in a controlled environment.	Easy method to use: requires only water samples, a temperature-controlled incubator and standard protocols for nutrient analyses.	Bulk nutrient analyses are not precise enough to detect small changes in relatively large nutrient pools. Therefore, the incubations have to be long.	(Seitzinger *et al.*, 2002; Lønborg and Alvarez-Salgado, 2012; Asmala *et al.*, 2013)
Isotope tracer	Single hours or days	A small amount of an isotope-labeled nutrient (tracer) is added. The extracellular turnover of the tracer is assumed to reflect the turnover of the ambient pool of nutrients to which the tracer is representative.	Uptake of the tracer is measured with high accuracy; even on short time-scales (h). Advanced applications of this method also allow assessing the fate of the tracer inside the cells.	It can be difficult to define the ambient nutrient pool to which the tracer is representative. For example the degree to which a specific organic N-containing molecule (urea or an amino acid) is taken up might not represent bioavailability of bulk DON.	(Boström *et al.*, 1988; Björkman and Karl, 2003; Bronk *et al.*, 2007; Kaplan *et al.*, 2008)
Regrowth	Ca. 4–7 days	The logistic growth of nutrient starved bacteria (or phytoplankton), utilizing a natural nutrient resource, is recorded. By determining the nutrient demand per unit growth, or the nutrient content per cell, the total bioavailability of the nutrient in question can be calculated from the total growth or cell yield.	Simple and straightforward method. Can be applied to measure bioavailability of multiple nutrients in parallel during short-term incubations.	The method is sensitive to variations in the net nutrient uptake per unit biological growth in the experiments. Additionally, if applied on DOC, the respiration during the experiments (and variations in growth efficiency) must be accounted for.	(Stepanauskas *et al.*, 2000b, Stepanauskas *et al.*, 2002)

Only methods that can be applied on multiple nutrients are included. Approaches which target only a certain type of nutrient, e.g. the oxygen consumption method to determine DOC bioavailability, are excluded.

**Table II:** Ranges of bioavailable fractions of dissolved organic carbon (DOC), dissolved organic nitrogen (DON), dissolved organic phosphorus (DOP) and total phosphorus (TP) reported in the literature, assessed using various methods

Study	DOC	DON	DOP^a^	TP	Method	System
(a) Bioavailability for natural bacterioplankton communities (dark incubations)						
Asmala *et al.* (2013)	0.08–0.11	0.05–0.22	–	–	Δ concentration	Finnish estuaries
Jansson *et al.* (2012)	–	–	–	0.03–0.43	Regrowth/Δ concentration	Boreal streams, seasonally
Kaplan *et al.* (2008)	0.08–0.24	–	–	–	Isotope tracer	Stream, range labile to semi-labile
Lønborg *et al.* (2009)	0.11–0.23	0.32–0.44	0.56–0.74	–	Δ concentration	Coastal upwelling, means ± SE
Lønborg and Alvarez-Salgado (2012)	0.02–0.51	0.10–0.65	0.30–0.96	–	Δ concentration	Coastal ocean review
Nausch and Nausch (2007)	–	–	0.33–0.60	–	Δ concentration	Baltic Sea basins
Petrone *et al.* (2009)	0.01–0.17	0.04–0.44	–	–	Δ concentration	Australian estuaries
Stepanauskas *et al.* (2000b)	–	0.19–0.55	–	–	Regrowth	Boreal streams
Stepanauskas *et al.* (2002)	–	0.08–0.72	0.04–1.3	–	Regrowth	Baltic Sea inlet river mouths
Wiegner and Seitzinger (2004)	0.07–0.32	n.d.–0.65	–	–	Δ concentration	Cedar bog wetland streams
Wiegner *et al.* (2006)	0.01–0.16	n.d.–0.40	–	–	Δ concentration	Eastern US rivers
Kaushal and Lewis (2005)	n.d.–0.30	0.15–0.71	–	–	Δ concentration	Montane streams (US)
(b) Bioavailability for plankton communities in light incubations						
Peters (1981)	–	–	–	0.19–0.83	Isotope tracer	Temperate lake and rivers
Seitzinger *et al.* (2002)	–	n.d.–0.73	–	–	Δ concentration	New Jersey runoff water

Since multi-element assessments are rare, the table includes all cases of multi-element macronutrient bioavailability measurements that could be found in the literature, of which two studies are from coastal oceans (Lønborg *et al.*, 2009; Lønborg and Alvarez-Salgado, 2012).

n.d., not detectable.

^a^DOP considered as dissolved TP-DRP, based on the assumption that DRP represents 100% of the inorganic part of dissolved TP.

## STOICHIOMETRIC CONTROLS: CONCEPTS, EMERGING FINDINGS AND PITFALLS

According to theory, basal productivity is regulated by, and hypothetically proportional to, the availability of the single nutrient which is in lowest supply relative to the biotic demand (i.e. Liebig's Law of the Minimum). When Redfield (Redfield, 1958) proposed that plankton communities use N_2_ gas fixation to autonomously adjust and optimize the N:P resource stoichiometry of the ocean (to ca. 16:1, by moles), P became a potential candidate for main limiting element, at least on long time-scales. Evidence for P as a limiting nutrient in freshwaters was presented in the 1970s through long-term whole-ecosystem experiments in the Experimental Lake Area, Canada (Schindler, 1977), indicating that productivity in eutrophied lakes (enriched with C, N and P in different combinations) could be mitigated by reducing the P supply, but not by decreasing the supply of any forms of C or N.

Today's plankton ecology has largely moved from the single-nutrient limitation paradigm to a so-called co-limitation paradigm, where the interacting (combined) influence of N, P and other nutrients is addressed (Sterner, 2008; Harpole *et al.*, 2011). This change is partly related to the recognition that different taxonomic and functional groups of planktonic producers have different requirements for bioavailable N and P, e.g. with low-resource N:P ratios often favored by fast-growing phytoplankton species (Hillebrand *et al.*, 2013). Thus, both N and P resource limitation can be expressed in parallel by different parts of the community. Further, the magnitude of N fixation rates has been found to differ greatly between systems and seasons, resulting in limited possibilities for N-fixing phytoplankton to compensate for low N:P resource ratios (Howarth and Marino, 2006; Marino *et al.*, 2006). Additional variability in N:P supply ratios, and thus changes in limitation, may be caused by seasonal shifts in terrestrial N demand and export to receiving lakes (e.g. Bergström *et al*., 2008), and/or by geographical patterns in anthropogenic atmospheric N deposition that can strongly influence patterns in nutrient limitation within and among regions (Bergström and Jansson, 2006; Elser *et al.*, 2009).

Owing to the development of the field of ecological stoichiometry (Sterner and Elser, 2002), data describing the absolute and relative pools of different nutrients (elements) has never played a more important role in plankton research than today. In this regard, analytical methods that allow for ecologically meaningful representation of the access to bioavailable nutrients is a prerequisite needed to further expand our understanding of aquatic productivity. The question is then: do the presently used standardized chemical nutrient analyses provide a sufficient representation of bioavailable nutrient pools?

### Pitfalls of bulk nutrient stoichiometry

#### Phytoplankton

Clear examples of the shortcomings of bulk nutrient stoichiometry are found in the literature related to PP regulation. For example, when Symons *et al.* (Symons *et al.*, 2012) surveyed a set of 21 subarctic lakes in Canada, it was hypothesized that phytoplankton growth would be limited by P alone, since the TN (total nitrogen):TP ratio in all lakes greatly exceeded the Redfield ratio. However, experiments in cubitainer enclosures showed P limitation on only five occasions; the chlorophyll-*a* production in the rest of the lakes was N-limited, co-limited, or not nutrient limited at all. The authors then made predictions of nutrient limitation patterns using ratios between organic or inorganic N and P fractions, but again these predictions failed in 71% of cases.

The experience from this Canadian study is not unusual. Bergström (Bergström, 2010) confirmed that the bulk TN:TP ratio failed to predict phytoplankton nutrient limitation in a review of alpine and boreal datasets in Europe and North America. In some studies, the predictability has increased if TN is replaced by dissolved inorganic N (DIN from NO_2_+NO_3_+NH_4_)- as the largest fraction of the TN in DOM-rich systems is DON, which is generally less bioavailable than the inorganic forms (Bergström, 2010)- and if the use of a theoretical N:P cutoff ratio is replaced by a statistical regression approach (Bergström, 2010; Kolzau *et al.*, 2014). In other studies, the latter approaches have also failed to predict nutrient limitation of PP (Symons *et al.*, 2012; Mischler *et al.*, 2014). These findings indicate that the ratios between commonly measured bulk nutrient fractions can differ systematically from the ratios of actual nutrient supply, very likely owing to differences in bioavailability.

#### Bacterioplankton

A common feature of inland waters is that large fractions of the N and P pools are associated with DOM of terrestrial origin. Within these organic pools, the proportion of C, N and P that is bioavailable to bacterioplankton can be extremely variable, ranging from undetectable to dominant (Seitzinger *et al.*, 2002; Stepanauskas *et al.*, 2002; del Giorgio and Davis, 2003). Therefore, the use of bulk nutrient fractions, again, leads to poor (or false) predictions of how productivity responds to nutrient loading in DOM-dominated waters. This problem was recently demonstrated in a recent study of bacterioplankton by Hitchcock and Mitrovic (Hitchcock and Mitrovic, 2013), where patterns of nutrient limitation of BP were analyzed in two Australian estuaries with C:N:P ratios of total resources within the proportions 732–5054:13–44:1. Considering bacterioplanktonic relative needs for different elements (Fagerbakke *et al.*, 1996; Vrede *et al.*, 2002), bulk resource stoichiometry would suggest primary P-limitation and secondary N-limitation. In contrast, according to the observations, BP was primarily C-limited, secondarily N-limited and only rarely influenced by P (Hitchcock and Mitrovic, 2013). In another study, N limitation of BP was observed with a TN:TP ratio as high as 66:1 (Berggren *et al.*, 2007), again questioning the view that bulk resource stoichiometry accurately predicts productivity.

## A NEW VIEW OF RESOURCE STOICHIOMETRY

### Bacterioplankton: emerging patterns from dark bioassays

We propose that to advance our general understanding of land–water interactions and nutrient dynamics in surface waters, there is a need to consider the size of ecologically relevant—bioavailable—pools of macronutrients (C, N and P). So far, bioavailability measurements have been sparsely applied, as they are perceived to be time-consuming and difficult to interpret, especially because bioavailability is an operational concept (del Giorgio and Davis, 2003). However, when compiling all available multi-element DOM bioavailability studies (Table II), including coastal studies, it becomes possible to speculate on general patterns in the bioavailability of different macronutrients for bacterioplankton.

One striking pattern that emerges for bacterioplankton is that, in virtually all studies of multi-element bioavailability, regardless of methodology, the values of bioavailable P are roughly twice as high as those for DON, which in turn are about twice as high as the bioavailability of DOC (Table II and references therein). Recently, the increasing bioavailability from C to N to P was confirmed in a unique large-scale review of hundreds of nutrient bioavailability assessments from coastal waters worldwide (Lønborg and Alvarez-Salgado, 2012), pointing to a general applicability of this pattern even in systems with much less land influence compared with inland waters. Little is known about the underlying chemistry that causes these differences, but compared with C, N appears to be more closely bound to bioavailable, non-humic components of the DOM pool (Kaushal and Lewis, 2005). Empirically, the pattern is also consistent with observations of increasing *in situ* turnover times from C to N to P in DOM-rich estuaries, indicating systematic patterns of reactivity across the different nutrient pools (Ziegler *et al.*, 2004). These systematic differences in bioavailability could also potentially explain many reported anomalies regarding the stoichiometric controls over BP, for example the previously mentioned nutrient limitation patterns observed by Hitchcock and Mitrovic (Hitchcock and Mitrovic, 2013).

Table II provides a temporally static depiction of relative bioavailability for C, N and P, but these properties likely vary at multiple time-scales. While extensive research has explored the seasonal controls over bulk nutrient exports across land–water boundaries (Lutz *et al.*, 2012; Sponseller *et al.*, 2014), few studies have addressed these dynamics for bioavailable pools *per se*. Importantly, the data that do exist suggests distinct seasonal trends for C, N and P that have a strong potential to drive temporal changes in the limitation of BP, but also likely of PP. For example several studies have shown peaks in bioavailable C for bacterioplankton during the spring snowmelt season, with subsequent declines during the summer (Kaushal and Lewis, 2005; Berggren *et al.*, 2009b).

Bioavailable organic N may show a similar seasonal peak in response to the spring flood (Stepanauskas *et al.*, 2000b; Berggren *et al.*, 2010a), but other studies suggest that this pool can remain elevated also during the growing season (Kaushal and Lewis, 2005), a period when inorganic N delivery to aquatic habitats is notoriously low (Bergström *et al.*, 2008; Sponseller *et al.*, 2014). However, in stark contrast to these patterns for C and N, Jansson *et al.* (Jansson *et al.* 2012) found that the absolute and relative concentrations of bioavailable P for bacterioplankton in boreal headwaters were low in spring, but increased several-fold in the transition to summer, before decreasing again in autumn, seemingly linked to temperature-dependent release of bioavailable P from soils.

### Bioassays for phytoplankton

Plankton ecologists have made significant progress in measuring the uptake of specific N- and P-containing biomolecules by phytoplankton, such as urea, amino acids (Bronk *et al.*, 2007) and nucleic acids (Muscarella *et al.*, 2014). However, these chemically defined bioavailable fractions most likely do not represent the relatively large total amounts of DON and DOP that have been consumed during incubations with phytoplankton in general (Peters, 1981; Seitzinger *et al.*, 2002; Korth *et al.*, 2012) and cyanobacteria in particular (Nausch and Nausch, 2007). This means that the development of the field is still dependent on bioavailability assessments also for phytoplankton.

Unfortunately, multi-element assessment of DOM bioavailability for phytoplankton is virtually absent from the scientific literature. However, it could be hypothesized the distinct between-element and seasonal patterns in N and P bioavailability for bacterioplankton are also going to affect nutrient limitation patterns in phytoplankton (Jansson, 1998) as well as the magnitude in overall basal productivity (Jansson *et al.*, 2003). Future research that describes spatial and temporal changes in bioavailable nutrient pools for phytoplankton, explores the mechanisms underlying these patterns, and evaluates the ecological influences of such dynamics, is likely to be particularly fruitful and challenging.

### A new view

**Fig. 1.**
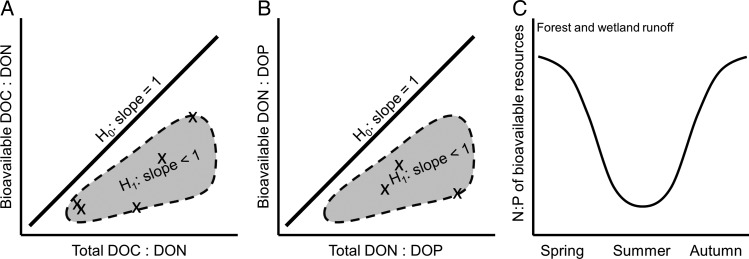
(**A** and **B**) Hypothetical relationships between nutrient ratios in bioavailable resources (for bacterioplankton, by moles) and the corresponding ratios among bulk organic nutrient resources. Symbols (x) show mean nutrient ratios (on relative scales) from different studies presented in Table II. (**C**) Hypothetical seasonal N:P pattern for bioavailable nutrients in runoff from natural terrestrial environments. H_0_, null hypothesis; H_1_, alternative hypothesis.

In spite the limitation in current knowledge, especially with regard to bioavailability for phytoplankton, there are strong reasons to question the assumption (or null hypothesis) that bulk DOM and bioavailable DOM generically have the same C:N:P stoichiometry. In fact, published studies to date for bacterioplankton rather support the alternative hypotheses that C:N (Fig. 1a) and N:P (Fig. 1b) are lower for bioavailable DOM fractions, compared with total DOM. Thus, with an increasing DOM dominance of surface waters, we can expect a greater discrepancy between the apparent DOC:TN:TP and the actual C:N:P ratios of the bioavailable nutrients. This means that BP in DOM-dominated systems can show C limitation (or co-limitation with N) even in cases where C:N:P ratios of the total nutrient resources are very high.

Further, the N:P of bioavailable nutrients in terrestrial source waters should be much lower in summer than other seasons (Fig. 1c). A succession of nutrient limitation of BP and PP from P in spring to N in summer is commonly reported for both lakes (Kolzau *et al.*, 2014) and estuaries (Conley, 1999; Hitchcock and Mitrovic, 2013). This pattern is usually explained by the temperature-boosted microbial sediment release of P during summer that coincides with reduced terrestrial exports of inorganic N. If similar mobilization of dissolved bioavailable P happens due to stimulated microbial soil organic matter processing during warm periods (Jansson *et al.*, 2012), then the runoff water from summer storms could exacerbate this switch toward N limitation in receiving waters in mid- to late summer. Furthermore, when terrestrial inputs of DON may be elevated, lakes affected by high-flow episodes in spring should switch toward temporary P limitation of BP (as indicated in Jansson *et al.*, 1999) and perhaps also of late spring PP.

## PROBLEMS, SOLUTIONS AND CLOSING REMARKS

In view of these emerging perspectives, increased use of bioavailability assessment is a most promising way to improve our understanding of nutrient control of basal productivity. However, there are problems associated with the practice of bioavailability measurements that are yet to be solved. In the remaining sections of the paper, we discuss some of the more important issues, and possible solutions.

### Taxa-dependent bioavailability: a problem?

Results from bacterial bioassays are traditionally interpreted as indicative of the general bioavailability of nutrients for biota, with relevance for natural bacterial and phytoplankton communities (Boström *et al.*, 1988; Stepanauskas *et al.*, 2000b). In support of such an assumption, bacterial communities across inland waters have similar capacity to degrade DOC (Comte and del Giorgio, 2011). Moreover, the bioavailability of land-derived organic N and P tends to be roughly the same for bacterial and phytoplankton communities (Nausch and Nausch, 2007; Korth *et al.*, 2012). Interestingly, although Korth *et al.* (Korth *et al.* 2012) found that DON of phytoplankton origin was re-assimilated more efficiently by the phytoplankton themselves than taken up by bacterioplankton, the uptake of land-derived DON was the same for bacterioplankton and phytoplankton. Thus, in the highly land-influenced DOM-dominated waters, it could be expected that nutrient bioavailability of DON and DOP is similar for the phytoplankton and bacterioplankton communities.

However, between-taxa similarities in bioavailability of land-derived DOM do not appear to apply to the largest (colloidal) fraction. Nutrient assimilation from this fraction rather appears highly taxa dependent (Fagerberg *et al.*, 2010) and regulated by specific uptake strategies, such as pinocytosis for dinoflagellates (Legrand and Carlsson, 1998) or surface interaction between colloids and bacteria, while, e.g. diatoms seemingly lack a corresponding direct uptake mechanism (Fagerberg *et al.*, 2010). Another complicating factor is that many forms of labile DON (e.g. amino acids) may serve primarily as an energy (i.e. C) or N source (Lutz *et al.*, 2011), and the circumstances under which these compounds are used by different groups of autotrophs and heterotrophs is uncertain (Bronk *et al.*, 2007). Clearly, to advance this field, more research is needed to support the assumptions invoked regarding patterns of bioavailability between different organism groups.

### Methodological limitations and the way forward

To overcome the problems of inconsistencies in methodology, standard multi-element bioavailability methods need to be developed. The only approach that has been applied simultaneously on organic fractions of C, N and P is the “Δ concentration” method (Table I), where measurements of the decline in bulk nutrient fractions are measured during long-term incubations (Lønborg *et al.*, 2009). However, unless the analytical precision of multi-element bulk organic nutrient analyses is substantially improved by future instrument development, there will be an inherent need for longer incubations that result in measurable declines in resource concentration, yet generate bioavailability values of questionable ecological relevance (Guillemette and del Giorgio, 2011). For example BP at a given moment will likely not be regulated by the availability of a particular form of C, N or P that could potentially be used within a 100-day time frame, which is a common time in bioavailability studies to date (Lønborg and Alvarez-Salgado, 2012).

Instead, we would like to highlight the short-term (4–7 days) regrowth bioassay approach as a promising alternative, where nutrient-dependent biological growth is used as a proxy for resource bioavailability (see Table I). This approach has successfully been applied in previous studies to simultaneously assess organic N and P bioavailability with a common methodological framework (Stepanauskas *et al.*, 2000a, 2002). It is also easy to apply both to bacterioplankton and phytoplankton (Table I). A new idea for future studies is to combine this method with bacterial growth efficiency measurements, which would make it possible to expand the assessment also to organic C bioavailability to bacterioplankon (Table I), allowing for the first-ever stoichiometric assessments of short-term bioavailability of most major macronutrients.

Further, from a stoichiometric standpoint, we argue that the focus of bioavailability assessments should be shifted toward that of total nutrient resource pools, rather than organic fractions only. In natural systems, the various forms of organic and inorganic N and P co-occur and are of course sampled together, and it is the amounts and stoichiometry of the total bioavailable resources that determine nutrient limitation patterns in plankton communities. Measurement of the total bioavailability has a particular advantage for P bioassays, since TP minus DRP, which is a common definition of DOP, is known to poorly represent organic P fractions in DOM-dominated waters (Jansson *et al.*, 2012). This means any DOP bioavailability assay which is dependent on DRP is potentially biased, but this problem of defining organic versus inorganic is avoided in assessment of TP bioavailability.

Once a methodological framework is established, we foresee a rapid development of the field, where changes in basal productivity in land-influenced systems can be increasingly predicted from changes in the environment. For this vision to be realized, we also need the synergetic effects of continued and expanded collaboration between ecosystem ecologists and organic chemists in possession of the now rapidly developing analytical tools to resolve the chemical composition of bioavailable fractions. However, as long as the major fractions of the bioavailable DOM pool remain chemically undefined, bioavailability assays are likely to serve as the most direct way to quantify true resource availability in aquatic systems.

### Concluding remarks

Our planet is currently undergoing rapid and diverse environmental changes that are altering the coupled cycles of C, N and P at global scales (Finzi *et al.*, 2011). These changes include regionally specific increases or decreases in anthropogenic N loading to ecological systems (Weyhenmeyer *et al.*, 2007), shifts in climate that potentially alter terrestrial productivity (Graven *et al.*, 2013) and, thus, nutrient demand, and increases in the mineralization and/or release of DOM from soils (Monteith *et al.*, 2007), all of which have the potential to alter the chemical conditions and multi-element balance of receiving waters. A major challenge for aquatic scientists is to predict how such changes will alter the productivity and trophic structure of lakes and estuaries in the future. Ecological stoichiometry provides an excellent foundation for making such predictions, but if not based on a biologically meaningful perspective on resource availability, then it will unlikely aid in our understanding of diverse future conditions. For example historical approaches to describing resource availability (e.g. using bulk TN:TP ratios) may be particularly poorly suited to help us understand the implications of brownification in surface waters currently observed across northern regions (Monteith *et al.*, 2007). We argue that a more explicit consideration of the biologically active resource pools to both heterotrophic and autotrophic elements of planktonic systems will provide a path toward a flexible and mechanistic understanding of aquatic ecosystem response to a range of potential environmental changes. Further, the refinement and use of multi-element bioassays appears to be one promising avenue toward this goal.

## FUNDING

We thank Beatrix Beisner for encouragement and invitation to write a Horizons paper. The Crafoord Foundation (grant #20120626), KSLA (grant #H13-0020-GBN) and Helge Ax:son Johnson's Foundation (grant #140622) provided funding of recent bioavailability research by the authors that has been the source of inspiration for this paper. M.B. was supported by the “Multistressor” strong research environment funded by FORMAS (grant #217-2010-126 to D.J. Conley *et al*.) and A.-K.B. by the FORMAS strong research environment grant #230-2010-67. Funding to pay the Open Access publication charges for this article was provided by Multistressor.
